# The association of multimorbidity and disability in a community-based sample of elderly aged 80 or older in Shanghai, China

**DOI:** 10.1186/s12877-016-0352-9

**Published:** 2016-10-27

**Authors:** Peng Su, Hansheng Ding, Wei Zhang, Guangfeng Duan, Yitong Yang, Rong Chen, Zengjie Duan, Lixia Du, Chunyan Xie, Chunlin Jin, Chaoqun Hu, Zixue Sun, Junrui Long, Lingling Gong, Wenhua Tian

**Affiliations:** 1 Department of Medical Administration, Guangzhou General Hospital of P.L.A., Guangzhou, 510010 China; 2 Shanghai Medical Science & Technology Information Center, Shanghai, 200031 China; 3 Shanghai Health Development Research Center, Shanghai, 200040 China; 4 Department of Health Services Management, the Second Military Medical University, Shanghai, 200433 China; 5 School of Statistics and Management, Shanghai University of Finance and Economics, Shanghai, 200433 China; 6 Shanghai Population and Development Research Center, Shanghai, 201199 China; 7 Community Health Service Center, Jiangning Street, Jing’an District, Shanghai, 200040 China; 8 School of Social Development and Public Policy, Fudan University, No. 220 Handan Rd, Shanghai, 200433 China

**Keywords:** Multimorbidity, Disability, Elderly, Community-dwelling, Chinese

## Abstract

**Background:**

Both multimorbidity and activities of daily living (ADL) disability and instrument activities of daily living (IADL) disability are common among elderly individuals. ADL/IADL disability may reduce individuals’ capacities for independent living and quality of life. This study aimed to examine the association between multimorbidity and ADL/IADL disability.

**Methods:**

A multi-stage cluster sample of 2058 residents aged 80 or older was investigated in Shanghai, China. Multimorbidity was defined as the simultaneous presence of two or more chronic diseases with ten common chronic conditions under consideration. Subjects who responded that they “need partial or full assistance” to any ADL/IADL items were defined as having ADL/IADL disability. We examined the association of multimorbidity with ADL/IADL disability, adjusted for socio-demographic characteristics by using logistic regression.

**Results:**

Of respondents, 23.23 % had ADL disability, 37.90 % had IADL disability, and 49.17 % had multimorbidity. After adjusted socio-demographic characteristics, a graded association was showed between ADL disability and the quantity of chronic conditions: odds ratio (OR) for 1 condition, 1.53(95 % confidence interval [CI], 1.04-2.24); OR for 2 conditions, 2.06(95 % CI, 1.43-2.96); OR for 3 conditions, 3.23(95 % CI, 2.14-4.86); OR for 4 or more conditions, 5.61(95 % CI, 3.26-9.66). Similar associations were also observed between the quantity of chronic conditions and IADL disability.

**Conclusions:**

The quantity of chronic conditions had relatively strong association with both ADL and IADL disability. Initiating prevention of additional chronic conditions and interventions on clusters of diseases may decrease the potential risk of ADL/IADL disability. Additionally, more attention should been given to the older low-income women living with relatives/non-relatives with multimorbidity.

## Background

Populations around the world are rapidly aging, and “healthy well” must be a global priority [1]. However, middle-income countries face more challenge of aging, since an unprecedented upward shift in life expectancy are witnessed among these countries [2]. According to data published by National Bureau of Statistics of China, 10.06 % of the total population were over 65 in 2014. But in Shanghai, this proportion was even up to 18.80 %. The change of age structure has important public health implications regarding the economic and social costs of chronic diseases [3, 4].

Studies have suggested that chronic diseases may result in disability in the elderly. Although disability can be defined in a number of ways, the activities of daily living (ADL) and instrument activities of daily living (IADL) are considered the most common ways [5]. Uddin et al. reported that hypertension was significantly associated with ADL/IADL disability [6], while Dunlop et al. found that older adults with diabetes or cerebrovascular disease were susceptible to have ADL/IADL disability [7].

As a result of increasing longevity, multiple comorbid conditions, or “multimorbidity”, have also become more common among elderly individuals [8, 9]. However, most previous studies focused on the association between a chronic disease and disability. Previous studies have demonstrated that multimorbidity is associated with negative health outcome, such as poor quality of life, disability, and mortality [10–12]. Even several studies suggested that the quantity of chronic diseases may be more closely associated with ADL disability among elderly adults [12–14]. Ralph et al. found the prevalence of ADL disability was graded increased with the quantity of chronic diseases [13]. Unfortunately, there were only three IADL included in Ralph study, so as to it definitely decreased power to imply the association between multimorbidity and IADL disability.

Most of previous studies were conducted in high-income countries, but social demographic status has been found strongly associated with the prevalence of multimorbidity and disability, regardless of whether social demographic status is measured by age, race, or area-based deprivation [13, 15]. There is a lack of a better understanding of the relationship between multimorbidity and disability among Asian elderly populations, as Asian was more susceptive to ADL disability compared with other races [13].

Since the proportion of elderly individuals aged 80 or older would increase as the aging process, and those people were more easily affected by chronic diseases [16]. Hence, the objective of this study was to examine the potential association between multimorbidity and ADL/IADL disability of elderly community-dwelling residents aged 80 years or older in Shanghai, China. We hypothesize that a positive graded association exists between the quantity of chronic conditions and likelihood of both ADL and IADL disability.

## Methods

### Data sources

This study used data of a large-scale survey initiated by Shanghai Health and Family Planning Commission from March to September 2013. This survey was designed as a multi-stage cluster study representative of the elderly population in Shanghai, China. In total, 8525 residents were investigated from 15 communities. Face-to-face interviews were conducted at participants’ homes by the staff of each surveyed neighborhood committee, each of which was trained by professional investigators from the workshop. Each participant completed the questionnaire independently. Detailed description of study, sample design, and quality control process is provided elsewhere [17]. In this study, we focused on individuals aged 80 or older, finally, 2058 residents aged 80 or older were included as our target population excluding those with missing data on ADL/IADL or chronic diseases.

All participants or their family members signed an informed consent (except for the illiterate, who only provided oral consent) and the study protocol received approval by the Ethics Committee of the Shanghai Medical and Technology Information Institute.

### Outcome variables

Our primary outcome variable of interest was ADL disability, as measured by self-report and investigators’ observation and judgment: bathing, dressing, toileting, transferring (getting in and out of bed or chair), eating, and getting around the home [13, 18]. As a secondary outcome variable, we examined IADL disability: using telephone, shopping, preparing meals, housekeeping (such as washing dishes or making the bed), doing laundry, using transport, medical care (such as taking medicines or pills) and financial management (such as budgeting, writing checks, paying rent and bills, and going to banks) [19]. Subjects who responded that they “need partial or full assistance” to any ADL/IADL items were defined as having ADL/IADL disability, while those who responded that they were “independent” in all ADL/IADL items were defined as no disability [13, 20, 21].

### Chronic conditions and multimorbidity

As most publications, multimorbidity is defined as the simultaneous presence of two or more chronic diseases in this study [8, 22–24]. On the spot, we used a questionnaire to collected data, and about the chronic diseases, we prepared a list of 17 common chronic diseases for help. According to the order of the list, we usually used two questions to certify the status of a certain chronic disease. For example, firstly, we would ask the participant “Do you suffer from hypertension?” If the participant answered “Yes”, then we would ask the second question “Is this result based on doctor diagnosed?” If the answer to the second question was still “Yes”, we concluded that this participant suffered from hypertension. In order to avoid missing chronic diseases information, finally, we would ask the participant “Do you suffer from any other chronic disease?” For this analysis, ten chronic conditions were included, namely hypertension, coronary heart disease, diabetes, cataract, cerebrovascular disease, dementia, chronic obstructive pulmonary disease (COPD), fracture, arthritis, and chronic kidney disease. Chronic conditions statuses, based on doctor’s diagnosis, were obtained through face-to-face interview.

### Social demographics

Social demographics included age, gender, income and living arrangement. Respondents were categorized into four age groups: 80 to 84, 85 to 89, 90 to 94, and 95 or older. Income was grouped into two categories: low (≤100 % of Shanghai basic cost of living allowances in 2013), and moderate (>100 % of the standard). Living arrangement was categorized as five groups: living alone, with spouse only, with spouse and children, with children only, and with other relatives/non-relatives.

### Statistical analysis

Analyses were performed using SAS statistical package 9.2 (SAS Institute Inc., 2007). After calculating descriptive statistics, bivariate analysis was used to examine the association of each socio-demographic factors and chronic diseases status with ADL/IADL disability in unadjusted models. Then, we used multiple logistic regression models to examine the association between multimorbidity and ADL/IADL disability after adjusting age, gender, income, and living arrangement.

## Results

### Sample characteristics

This study included 2058 participants. Overall, 23.23 % of respondents reported ADL disability and 37.90 % reported IADL disability. As Table 1 showed, more than half individuals were women aged 80 to 84, and 96.60 % had moderate income. The percentage of population living with children only was 38.19 %, followed by living with spouses only (29.66 %) and living alone (21.23 %). Of this group individuals, 62.00 % had hypertension, 30.56 % coronary heart disease, 19.92 % diabetes, 14.09 % cataract, 11.18 % cerebrovascular disease, 6.03 % arthritis, 2.43 % fracture, 2.33 % dementia, 1.85 % chronic kidney disease, and 1.85 % COPD. Totally, 77.99 % participants reported at least one diagnosed condition, and 49.17 % reported multimorbidity.

**Table 1:** Characteristics of community-dwelling individuals in 2013 in Shanghai, China

Characteristics	N (%)
Age, y	
80–84	1198 (58.21)
85–89	578 (28.09)
90–94	217 (10.54)
95+	65 (3.16)
Gender	
Male	867 (42.13)
Female	1191 (57.87)
Income^a^	
Moderate	1849 (96.60)
Low	65 (3.40)
Living arrangement	
Living alone	428 (21.23)
With spouse only	598 (29.66)
With spouse and children	161 (7.99)
With children only	770 (38.19)
With other relatives/non-relatives	59 (2.93)
Number of Chronic Diseases	
0	453 (22.01)
1	593 (28.81)
2	632 (30.71)
3	284 (13.8)
4+	96 (4.66)
Multimorbidity	2012 (49.17)
ADL disability	478 (23.23)
IADL disability	780 (37.90)

*Abbreviations*: *N* number, *y* year

^a^Income was grouped into two categories: low (≤100 % of Shanghai basic cost of living allowances in 2013), and moderate (>100 % of the standard)

### Associations between ADL/IADL disability and multimorbidity

As Table 2 showed, in unadjusted models, having multimorbidity was significantly associated with ADL disability, but not those having 1 condition. After adjusting age, sex, income, all four groups were significantly and independently associated with ADL disability (OR (odds ratio) for 1 condition group = 1.53, 95 % CI (confidence intervals) =1.04-2.24; OR for 2 conditions group = 2.06, 95 % CI = 1.43-2.96; OR for 3 conditions group = 3.23, 95 % CI = 2.14-4.86; OR for 4+ conditions group = 5.61, 95 % CI = 3.26-9.66).

**Table 2:** Association between multimorbidity and activities of daily living disability for community-dwelling individuals in 2013 in Shanghai, China^a^

Characteristics	N (%)	Unadjusted OR (95 % CI)	*p*	Adjusted OR (95 % CI)	*p*
Age, y					
80–84	176 (8.55)	1 [Reference]			
85–89	162 (7.87)	2.26 (1.78-2.88)	<.001	2.00 (1.53-2.61)	<.001
90–94	104 (5.05)	5.34 (3.92-7.29)	<.001	5.23 (3.66-7.48)	<.001
95+	36 (1.75)	7.21 (4.31-12.06)	<.001	7.66 (4.14-14.19)	<.001
Gender					
Male	175 (8.50)	1 [Reference]			
Female	303 (14.72)	1.35 (1.09-1.67)	.005	1.17 (0.91-1.5)	.23
Income^b^					
Moderate	417 (21.79)	1 [Reference]			
Low	26 (1.36))	2.29 (1.38-3.81)	.001	1.11 (0.62-1.99)	.73
Living arrangement					
Living alone	60 (2.98)	1 [Reference]			
With spouse only	101 (5.01)	1.25 (0.88-1.76)	.21	1.70 (1.15-2.50)	.007
With spouse and children	39 (1.93)	1.96 (1.25-3.08)	.003	2.39 (1.46-3.91)	<.001
With children only	230 (11.41)	2.61 (1.91-3.57)	<.001	2.28 (1.61-3.21)	<.001
With other relatives/non-relatives	33 (1.64)	7.78 (4.35-13.93)	<.001	6.07 (3.18-11.59)	<.001
Number of Chronic Diseases					
0	64 (3.11)	1 [Reference]			
1	109 (5.30)	1.37 (0.98-1.92)	.07	1.53 (1.04-2.24)	.03
2	154 (7.48)	1.96 (1.42-2.7)	<.001	2.06 (1.43-2.96)	<.001
3	107 (5.20)	3.67 (2.57-5.25)	<.001	3.23 (2.14-4.86)	<.001
4+	44 (2.14)	5.14 (3.18-8.32)	<.001	5.61 (3.26-9.66)	<.001

*Abbreviations*: *N* number, *y* year, *OR* odds ratio, *CI* confidence interval

^a^Adjusted for age, gender, income and living arrangement

^b^Income was grouped into two categories: low (≤100 % of Shanghai basic cost of living allowances in 2013), and moderate (>100 % of the standard)

As Table 3 showed, in unadjusted models, all groups were significantly associated with IADL disability. After adjusting age, sex, income, the association is still significant and independent (OR for 1 condition group = 1.51, 95 % CI =1.10-2.08; OR for 2 conditions group = 2.16, 95 % CI = 1.58-2.94; OR for 3 conditions group = 2.96, 95 % CI = 2.05-4.26; OR for 4+ conditions group = 5.51, 95 % CI = 3.23-9.4).

**Table 3:** Association between multimorbidity and instrument activities of daily living disability for community-dwelling individuals in Shanghai, China^a^

Characteristics	N (%)	Unadjusted OR (95 % CI)	*p*	Adjusted OR (95 % CI)	*p*
Age, y					
80–84	307 (14.92)	1 [Reference]			
85–89	276 (13.41)	2.65 (2.15-3.27)	<.001	2.41 (1.91-3.04)	<.001
90–94	144 (7.00)	5.73 (4.2-7.81)	<.001	5.5 (3.82-7.91)	<.001
95+	53 (2.58)	12.82 (6.76-24.31)	<.001	11.89 (5.48-25.82)	<.001
Gender					
Male	274 (13.31)	1 [Reference]			
Female	506 (24.59)	1.60 (1.33-1.92)	<.001	1.36 (1.09-1.7)	.009
Income^b^					
Moderate	689 (35.53)	1 [Reference]			
Low	46 (2.40)	4.16 (2.42-7.16)	<.001	1.94 (1.04-3.63)	.04
Living arrangement					
Living alone	98 (4.86)	1 [Reference]			
With spouse only	156 (7.74)	1.19 (0.89-1.59)	.04	1.83 (1.31-2.55)	<.001
With spouse and children	67 (3.32)	2.4 (1.63-3.53)	<.001	3.37 (2.18-5.2)	<.001
With children only	396 (19.64)	3.57 (2.73-4.65)	<.001	3.33 (2.47-4.5)	<.001
With other relatives/non-relatives	43 (2.13)	9.05 (4.89-16.77)	<.001	8.05 (4.1-15.82)	<.001
Number of Chronic Diseases					
0	114 (5.54)	1 [Reference]			
1	191 (9.28)	1.41 (1.08-1.86)	.01	1.51 (1.10-2.08)	.01
2	263 (12.78)	2.12 (1.63-2.76)	<.001	2.16 (1.58-2.94)	<.001
3	150 (7.29)	3.33 (2.43-4.56)	<.001	2.96 (2.05-4.26)	<.001
4+	62 (3.01)	5.42 (3.39-8.67)	<.001	5.51 (3.23-9.40)	<.001

*Abbreviations*: *N* number, *y* year, *OR* odds ratio, *CI* confidence interval

^a^Adjusted for age, gender, income and living arrangement

^b^Income was grouped into two categories: low (≤100 % of Shanghai basic cost of living allowances in 2013), and moderate (>100 % of the standard)

### Associations between ADL/IADL disability and socio-demographic characteristics

Age was significantly associated with ADL disability in both unadjusted model and adjusted model, and the OR was raised sharply as the growth of the age. Difference by age for IADL disability was larger than ADL disability, but they were in the same direction. Female and low income were significantly associated with ADL/IADL disability in unadjusted models, but after adjusting socio-demographic characteristics, Female and low income were only significantly associated IADL disability. Compared with participants living alone, all other living arrangements were significantly associated with ADL/IADL disability, and those living with other relatives/non-relatives had the highest risk (OR for ADL disability = 6.07, 95 % CI = 3.18-11.59; OR for IADL disability = 8.05, 95 % CI = 4.1-15.82).

## Discussion

As studies implied, the association between specific chronic diseases and ADL/IADL disability may vary, such as diabetes, hypertension, and dementia [8, 20, 25]. This study demonstrates a consistent and graded association between the quantity of chronic conditions and the likelihood of disability in either ADL or IADL, despite the heterogeneity of chronic conditions, or the severity of the conditions. Our findings were consistent with previous studies, and also were the supplement to these studies. In Arokiasamy’s study and Ralph’s studies, they both found that the association between chronic conditions and ADL disability was stronger as the increase of the quantity of chronic conditions [13, 23]. Unfortunately, as there was no IADL items included in Arokiasamy’s study, and only three IADL items were included in Ralph’s studies, the risk of the quantity of chronic conditions to IADL disability was not implied clearly. In our study, we included all eight IADL items, and firstly certified that the association between chronic conditions and IADL disability was similar to ADL disability. For example, OR of more than four chronic conditions for ADL disability = 5.61, and OR of more than four chronic conditions for IADL disability = 5.51.

A systematic review of studies on multimorbidity suggests different operational definitions of multimorbidity might influence the burden or impacts of multimorbidity [22]. But, the size and concrete chronic diseases were different between different researches. For example, in Ralph’s study, they included five common chronic diseases (arthritis, osteoporosis, hypertension, hypercholesterolemia, and diabetes) [13]; in Taylor’s study, they included 7 conditions (diabetes, asthma, COPD, cardio-vascular disease, osteoporosis, arthritis and mental health) [24]; in Wister’s study, they included 19 physical chronic conditions (arthritis, asthma, back problems, blood pressure, bronchitis, cancer, cataracts, COPD, diabetes, emphysema, glaucoma, heart disease, migraine headaches, osteoporosis, stroke, thyroid condition, ulcers, and urinary incontinence) [8]. According to previous studies and group discussions, 10 chronic diseases were included in this study. And the prevalence of multimorbidity in our study was 49.17 %. In a systematic review, the prevalence of multimorbidity in older persons ranged from 55 to 98 %[26]. Therefore, the prevalence of multimorbidity was relatively low. There may be some reasons. Firstly, different studies used different definitions and even in using the same definition, the size and concrete chronic diseases may also different. In our study, we only included physical chronic conditions, but in some studies, mental health problems might be included. Secondly, the criteria of having chronic conditions or not was different. In this study, chronic conditions were determined by self-report (ever been told by a doctor). Therefore, those individuals, who had chronic conditions but not yet accepted the doctors’ diagnosis, were excluded from multimorbidity group.

In addition, primary health care general practitioners take most of the responsibility for prevention and treatment of chronic diseases in China. Since the chronic conditions included in this study were treated for the most part by primary health care general practitioners. Findings from this study confirm the importance of developing and providing interventions for managing multimorbidity and preventing additional chronic conditions to reduce potential for ADL/IALD disability among elderly individuals in community settings. Based on our findings, we suggest that primary health care and public health practitioners should pay more attention on managing multimorbidity and preventing additional chronic conditions among community-dwelling elderly population.

In addition, other researches on patterns of chronic conditions also support the importance of recognizing multimorbidity in other aspects: firstly, multimorbidity is increasingly prevalent among older population all over the world [26]; secondly, multimorbidity patients represent an increasing proportion that are associated with increased health care cost and utilization [27]; thirdly, interventions that focus on clusters of diseases instead of a single disease are more efficient [13].

The relationship between socio-demographic characteristics was partly consisted with previous studies. As we all knew, advanced age is probably one of the most important factor related to functional decline, even some studies suggest the increase in relative risk of functional loss is about 2.0 for each 10-year increase in age [28]. However, in Formiga’s study, age lost the association with ADL disability in nonagenarian population [29]. In this study, we demonstrated a consistent and graded association between the increase of age and likelihood of disability in ADL/IADL just like the quantity of chronic conditions, and exponential relationship even displayed a dramatic increase among nonagenarian population. Different results may due to differences in sample sizes (282 vs. 97).

Living arrangement was associated with ADL/IADL disability in previous studies, this relationship also confirmed in this study [15]. Elderly people living alone appeared to have better functionality, and those living with other relatives/non-relatives have the highest risk of ADL/IADL disability. In adjusted models, gender and income were not significantly associated with ADL disability, but low-income women were more likely to have IADL disability. It is partly because the prevalence of IADL disability was generally higher than ADL in this group of individuals; additionally, disability in IADL might be a more sensitive predictor of disability than ADL [30].

This study has several strengths. Firstly, the prevalence of multimorbidity reported in this study was based on a list of chronic conditions that included the most common conditions among a large representative community sample; thus our findings provided representative fundamental data for horizontal comparison between different countries and regions. Secondly, our findings demonstrated consistent and graded association between likelihood of disability in ADL/IADL and the quantity of chronic conditions. These findings mean that the differences of the heterogeneity or severity of chronic conditions couldn’t conceal the impact of the quantity of chronic conditions. To primary health care and public health practitioners, these information reminded that the individuals with mild chronic conditions also should been pay more attention. To the best of our knowledge, our study is the first to focus on the association between multimorbidity and ADL/IADL disability among the community-dwelling oldest-old population, especially including all eight IADL items. Thirdly, we found the association between socio-demographic characteristics and ADL/IADL disability that the low-income women living with relatives/non-relatives were more likely to experience ADL/IADL disability. And these findings provided simple and useful information for primary health care general practitioners and public health workers to screen for those at high risk of ADL/IADL disability among the elderly. As health resources are precious and finite, they should be used more specifically and efficiently.

Our study has several limitations. Because the sample was cross-sectional, we were not able to identify the causation between multimorbidity and onset of ADL/IADL disability. However, our findings provide new data to support earlier researches that show multimorbidity may be an important factor of ADL disability, and our findings extend this association from ADL to IADL [13, 14]. Only limited socio-demographic characteristics were available. Some characteristics, such as education, marital status, that were clearly associated with ADL or IADL disability, were not included in this survey [31]. As the starting point of our study was that the health conditions or diseases had already formed, we regretted our inability to do further research on preventing chronic diseases, and the exact chronic prevention steps will be the next aim of our study.

## Conclusions

This study demonstrates that, the quantity of chronic conditions has relatively strong association with both ADL and IADL disability. Initiating prevention of additional chronic conditions and interventions on clusters of diseases may decrease the potential risk of ADL/IADL disability. Considering the age of the objective population, all these measures should been planned and in taken in advance. Additionally, more attention should been given to the older low-income women living with relatives/non-relatives with multimorbidity.

## Abbreviations

ADL: Activities of daily living
CI: Confidence interval
COPD: Chronic obstructive pulmonary disease
IADL: Instrument activities of daily living
OR: Odds ratio
